# Elevation of cardiac troponin I during non-exertional heat-related illnesses in the context of a heatwave

**DOI:** 10.1186/cc9034

**Published:** 2010-05-27

**Authors:** Pierre Hausfater, Benoît Doumenc, Sébastien Chopin, Yannick Le Manach, Aline Santin, Sandrine Dautheville, Anabela Patzak, Philippe Hericord, Bruno Mégarbane, Marc Andronikof, Nabila Terbaoui, Bruno Riou

**Affiliations:** 1 Emergency department Hôpital Pitié-Salpêtrière et Université Pierre et Marie Curie-Paris 6, 47-83 boulevard de l'hôpital, 75651 Paris Cedex 13, France; 2 Emergency department Hôpital Bicêtre, 78 rue du général Leclerc 94275 Le Kremlin Bicêtre Cedex, France; 3 Department of Anesthesiology and Critical Care Hôpital Pitié-Salpêtrière et Université Pierre et Marie Curie-Paris 6, 47-83 boulevard de l'hôpital, 75651 Paris Cedex 13, France; 4 Emergency department Hôpital Henri Mondor 51 avenue du maréchal de Tassigny 94010 Créteil Cedex, France; 5 Emergency department Hôpital Tenon 4 rue de la Chine 75970 Paris Cedex 20; 6 Emergency department Hôpital Européen Georges Pompidou, 20 rue Leblanc 75908 Paris Cedex 15, France; 7 Emergency department Hôpital Saint-Antoine 184 rue du faubourg Saint-Antoine 75571 Paris Cedex 12, France; 8 Department of Critical Care Medicine Hôpital Lariboisière 2 rue Ambroise Paré 75475 Paris Cedex 10, France; 9 Emergency department Hôpital Antoine Béclère 157 rue de la porte de Trivaux 92141 Clamart Cedex, France; 10 Emergency department, Hôpital Bichat-Claude Bernard 46 rue Henri Huchard, Assistance-Publique Hôpitaux de Paris, 75018 Paris, France

## Abstract

**Introduction:**

The prognostic value of cardiac troponin I (cTnI) in patients having a heat-related illness during a heat wave has been poorly documented.

**Methods:**

In a post hoc analysis, we evaluated 514 patients admitted to emergency departments during the August 2003 heat wave in Paris, having a core temperature >38.5°C and who had analysis of cTnI levels. cTnI was considered as normal, moderately elevated (abnormality threshold to 1.5 ng.mL^-1^), or severely elevated (>1.5 ng.mL^-1^). Patients were classified according to our previously described risk score (high, intermediate, and low-risk of death).

**Results:**

Mean age was 84 ± 12 years, mean body temperature 40.3 ± 1.2°C. cTnI was moderately elevated in 165 (32%) and severely elevated in 97 (19%) patients. One-year survival was significantly decreased in patients with moderate or severe increase in cTnI (24 and 46% vs 58%, all *P *< 0.05). Using logistic regression, four independent variables were associated with an elevated cTnI: previous coronary artery disease, Glasgow coma scale <12, serum creatinine >120 μmol.L^-1^, and heart rate >110 bpm. Using Cox regression, only severely elevated cTnI was an independent prognostic factor (hazard ratio 1.93, 95% confidence interval 1.35 to 2.77) when risk score was taken into account. One-year survival was decreased in patients with elevated cTnI only in high risk patients (17 vs 31%, *P = *0.04).

**Conclusions:**

cTnI is frequently elevated in patients with non-exertional heat-related illnesses during a heat wave and is an independent risk factor only in high risk patients where severe increase (>1.5 ng.mL^-1^) indicates severe myocardial damage.

## Introduction

In contrast to exertional heatstroke related to a high production of heat during strenuous exercise, non-exertional or classic heatstroke results from prolonged exposure to high temperature [1]. Classic heatstroke is encountered in tropical areas, but exceptional heat waves have been increasingly reported in temperate countries [2-4], and are possibly related to climate change [5]. The health consequences of these heat waves can be catastrophic leading to overcrowding of health facilities [6], excess mortality [7] and poor long-term outcome in surviving patients [8-11].

We have recently conducted an observational study of patients admitted to an emergency department (ED) during the French heat wave which occurred in August 2003, and identified several risk factors associated with mortality [11]. Knowledge of these risk factors is important since a heat wave is a catastrophic event leading to considerable overload in ED [6] and determining the therapeutic priorities, including access to the ICU appears essential. In that study, we also suggested that, during a heat wave, extended criteria of elevated core temperature should be used because of the considerable excess mortality encountered in an elderly population [6,7]. In an important subgroup of our patients, cardiac troponin I (cTnI) in serum was measured. Heatstroke has been very rarely reported as a possible cause of elevation of cTnI [11-15], although heat wave as been shown to be associated with an increased risk of sudden cardiac death [16]. Recent studies of cases of severe heatstroke admitted to ICU have suggested that such elevation might be observed as part of an early multiple organ dysfunction and might be associated with poor outcome [15].

Thus we performed a post hoc analysis of patients admitted to ED during the French heat wave of 2003 and in whom cTnI levels were measured on admission. As a primary end point, we assessed whether an increased cTnI could be an independent prognostic factor during heat-related illnesses. We also assessed the incidence and severity of cTnI elevation and looked at variables associated with such elevation.

## Materials and methods

This was an ancillary study of a multi-center cohort-study of hyperthermic patients admitted to 16 EDs belonging to the teaching hospital network of the Paris area (Assistance Publique-Hopitaux de Paris, Paris, France) during the heat wave of August 2003 in France [11]. This study was authorized by the Conseil National Informatique et Libertés (CNIL, Paris, France) and approved by the ethical committee of our hospital (Comité de Protection des Personnes Pitié-Salpêtrière, Paris, France) which waived the requirement for informed consent. The criteria for inclusion in the study were: 1) emergency admission in the adult ED of one of the participating centers between 5 August and 14 August 2003; 2) core temperature ≥38.5°C. However, we also studied a subgroup of patients with a core temperature ≥40°C. The study period covered the core period of the heat wave and of excess short-term mortality rate recorded during this time [6,8]. There were no exclusion criteria, except age <16 years. In the present ancillary study, measurement of cardiac troponin I at admission was used as an additional inclusion criterion.

An electronic clinical record form was used to collect data (Télémédecine Technologies, Boulogne, France). Data entered in the database were verified by on site clinical monitoring. Inconsistency between data was systematically checked and solved. The complete chart was examined by an expert panel who decided if the patient had critically-ill conditions that might have required admission to the ICU. To assess dependency in this elderly population, the validated Activities of Daily Living (ADL) scale was recorded [17]. The ADL scale ranges from 0 (worse) to 6 (best, autonomy free). Patients were followed until death or until one year after admission to the ED. Surviving patients or their family were contacted and interviewed by telephone. If contact could not be made, tracking was attempted through health care providers, particularly general practitioners, or any acquaintances identified in the medical record. When patients were lost to follow-up, an inquiry was sent to the French national registry of death (Institut National de la Statistique et des Etudes Economiques, Paris, France) in order to obtain information concerning the fatality.

We recently developed a heatstroke risk score using nine independent prognostic factors. To develop this score, we assigned the prognostics factors identified by multivariate analysis using only variables available at admission and using weighted points proportional to the β regression coefficient values and rounded to the nearest integer, as follows: previous treatment with diuretics (1 point), living in an institution (1 point), age >80 years (1 point), cardiac disease (1 points), cancer (2 points), core temperature >40°C (2 points), systolic arterial pressure <100 mmHg (4 points), Glasgow coma scale <12 (5 points), and transportation to hospital by ambulance (5 points). We defined three risk groups: low (0 to 6 points), intermediate (7 to 12 points), and high risk (13 to 22 points) for death [11]. In some patients, despite some missing values that precluded exact calculation of the risk score, the risk score category could be allocated because these missing values did not modify it.

### Measurement of cardiac troponin I

Because measurement of cTnI in serum was performed in different centers, assays were performed using different apparatus. The following apparatus were used with their associated values of detection threshold and abnormality threshold (different values could have been used in different centers): Opus, Dade Behring, Paris La Défense, France (0.04 and 0.15 ng.ml^-1^), RXL, Dade Behring (0.01 and 0.15 ng.ml^-1^; 0.015 and 0.15 ng.ml^-1^), SCS Dade Behring (0.03 and 0.15 ng.ml^-1^), ACS 180, Bayer Diagnostic, Puteaux, France (0.10 and 0.20 ng.ml^-1^; 0.10 and 0.50 ng.ml^-1^), Centaur, Bayer Diagnostic (0.10 and 0.15 ng.ml^-1^), Access 2, Beckman Coulter, Fullerton, CA, USA (0.01 and 0.05 ng.ml^-1^; 0.01 and 0.15 ng.ml^-1^;) AXSYM Abbot Laboratories, Abbot Park, IL, USA (0.024 and 0.04 ng.ml^-1^), Vitros ECI, Ortho-Clinical Diagnostics, Rochester, NY, USA (0.04 and 0.08 ng.ml^-1^) A value above the abnormality threshold was considered as indicating myocardial damage. A value >1.5 ng.L^-1 ^was considered as indicating severe myocardial damage, as previously reported [18].

### Statistical analysis

Data are expressed as mean ± standard deviation (SD), median and its 25 to 75 interquartile for non Gaussian variables (Kolmogorov-Smirnov test), or number and percentage. Comparison of two groups was performed using the Student *t *test, the Mann-Whitney U test, and Fisher's exact method when appropriate. We also performed a multiple backward logistic regression to assess variables associated with an elevation of cTnI and calculated their odds ratio and 95% confidence interval. To avoid overfitting, we used a conservative approach and included only the significant variables in the univariate analysis (*P *value of entry ≤0.10), except for some variables which were thought to be associated with an increase in cTnI or had been demonstrated to be prognostic in our previous studies [11]. If the Pearson correlation coefficient between variables was 0.60 or more, only the variable judged to be clinically more relevant was entered into the multivariate model. Continuous variables were transformed in dichotomous variables, using receiver-operating characteristic (ROC) curves, the threshold being that which minimizes the distance to the ideal point (1 = sensitivity = specificity), as previously described [19]. The discrimination of the final model was assessed using the C statistic and its calibration using the Hosmer-Lemeshow statistic. Survival was estimated by the Kaplan-Meier method, and differences in survival between groups were assessed by the log-rank test. For multiple comparisons, the Bonferroni correction was applied. To verify that cTnI is an independent risk factors, multivariate Cox proportional-hazards model was used to determine the contribution of cTnI and our risk score expressed as three levels (low, intermediate, and high-risk score) [11]. We also analyzed the subgroup of patients with core temperature >40°C.

All statistical tests were two-sided, and a *P *value of less than 0.05 was required to reject the null hypothesis. Statistical analysis was performed using NCSS 2001 software (Statistical Solutions Ltd, Cork, Ireland).

## Results

Among the 1,456 patients included in the core study, cTnI levels were measured in 514 (35%) patients who constituted the study sample. Patients in this sample were older (84 ± 12 vs 76 ± 12 years, *P *< 0.001), had a highest body temperature (40.3 ± 1.2 vs 39.9 ± 1.1°C, *P *< 0.001), a lower Glasgow coma scale (14 (9 to 15) vs 15 (13 to 15), *P *< 0.001), a lower ADL score (5 (2 to 6) vs 6 (2 to 6), *P *< 0.01), had a greater incidence of self-reported cardiac disease (36 vs 20%, *P *< 0.001) and were more frequently transported to the hospital by an ambulance (96 vs 86%, *P *< 0.001). They were also more frequently considered as critically ill by the independent expert panel (36 vs 22%, *P *< 0.001) and their survival rate at one year was lower (48 vs 61%, *P *< 0.001).

During up to one year of follow up, 40 (8%) patients entering the study were lost to follow-up. In the multivariate analysis, two variables were significantly associated with a loss to follow-up: a Glasgow coma score of 15 (odds ratio 4.77, 95% CI 1.72 to 13.21, *P *= 0.003), and lack of any pre-existing disease (odds ratio 3.03, 95% CI 1.40 to 6.54, *P *< 0.005).

**Figure 1:**
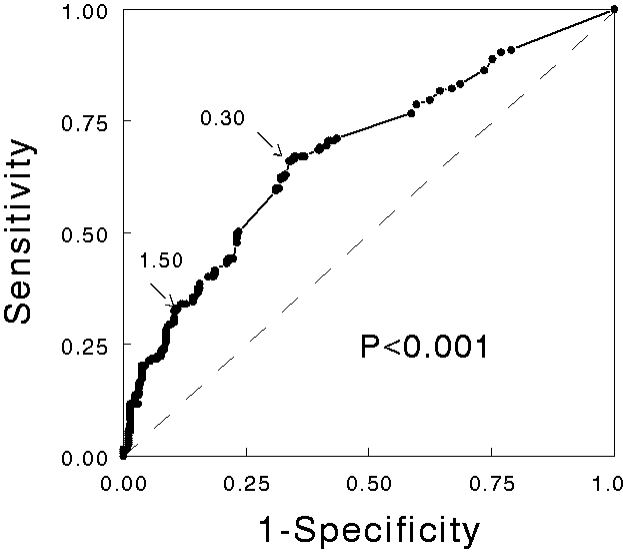
**Receiver-operating curve showing the relationship between cardiac troponin I elevation and death (n = 514)**. The best threshold (0.30 ng.mL^-1^) was associated with a sensitivity of 0.66 and a specificity of 0.66 (arrow). The threshold retained to define severe myocardial damage (1.50 ng.mL^-1^; see Methods) was associated with a sensitivity of 0.33 and a specificity of 0.89 (arrow). The area under the ROC curve was 0.68 (95% confidence interval 0.63 to 0.73) and was significantly different (*P *< 0.001) from the no discrimination curve (dotted line).

**Figure 2:**
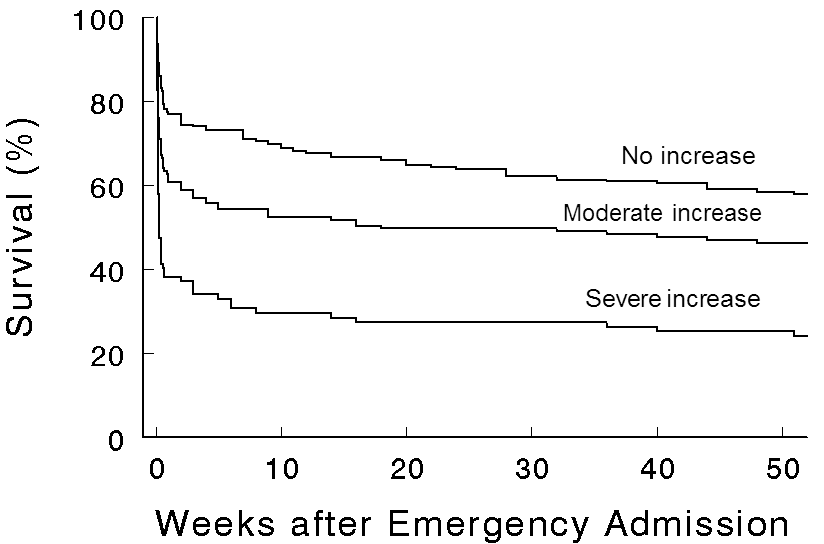
**Kaplan-Meier Survival Curves in patients, according to troponin elevation ranges**. Without elevation of cardiac troponin I (cTnI) (n = 252), moderate increase (abnormality threshold to 1.5 ng.mL^-1^, n = 165) and severe increase in cTnI (>1.5 ng.mL^-1^, n = 97). All differences were significant (*P *< 0.05).

Among the 514 patients where levels were measured, cTnI was elevated in 268 (52%) patients, and severe myocardial damage was observed in 97 (19%) patients. The comparison of patients with and without cTnI elevation is shown in Table 1. The incidence of elevated cTnI was higher in patients admitted into ICU (74 vs 49%, *P *= 0.005) and in critically ill patients (74 vs 38%, *P *< 0.001). The ROC curve indicates that cTnI was significantly associated with the prediction of death (Figure 1). The survival rate was significantly lower in patients with elevated cTnI (Figure 2). In the multivariate analysis, four variables were independently associated with an elevation in cTnI: preexisting self-reported coronary artery disease, creatinine >120 μmol.L^-1^, Glasgow coma scale <12 and heart rate >110 bpm (Table 2).

**Table 1:** Comparison of patients with normal and elevated cardiac troponin I (cTnI)

Variable	Normal cTni (n = 252)	Elevated cTnI (n = 62)	*P*	% missing g values*
Age (years)	83 ± 11	85 ± 12	0.18	0%
Age >80 years	179/252 (71)	199/262 (76)	0.23	0%
Male sex	109/252 (43)	87/262 (33)	0.02	0%
Katz index	6 (3 to 6)	5 (2 to 6)	0.006	20.4%
Living in an institution	48/252 (19)	63/262 (24)	0.24	0%
Transportation to the hospital in an ambulance	241/252 (96)	253/262 (96)	1.00	0%
*Pre-existing disease*				
Hypertension	100/249 (40)	86/256 (33)	0.14	1.8%
Coronary artery disease	57/249 (23)	89/256 (35)	0.004	1.8%
Heart failure	29/249 (12)	30/256 (12)	1.00	1.8%
Diabetes	32/249 (13)	35/256 (14)	0.79	1.8%
Cancer	30/249 (12)	12/256 (5)	0.003	1.8%
*Chronic medications*				
Diuretics	85/247 (34)	92/247 (37)	0.57	3.9%
CEI or AII I	51/247 (21)	60/247 (24)	0.39	3.9%
Beta-blockers	39/247 (16)	27/247 (11)	0.15	3.9%
Calcium inhibitors	38/247 (15)	45/247 (18)	0.47	3.9%
Nitrates	35/247 (14)	61/247 (24)	0.004	3.9%
Inotropic drugs	25/247 (10)	35/247 (14)	0.21	3.9%
Antiarrhythmic drugs	33/247 (13)	19/247 (8)	0.06	3.9%
Anticoagulant or antiaggregants	110/247 (44)	101/247 (41)	0.47	3.9%
Statines	20/247 (8)	14/247 (6)	0.37	3.9%
*Clinical signs*				
Core temperature (°C)	39.9 ± 1.1	40.6 ± 1.3	<0.001	0%
Core temperature >40°C	105/252 (42)	176/262 (67)	<0.001	0%
SBP (mmHg)	120 ± 37	104 ± 42	<0.001	0%
SBP <100 mmHg	31/214 (12)	74/254 (29)	<0.001	0%
Glasgow coma scale	15 (13 to 15)	12 (6 to 15)	<0.001	10.0%
Glasgow coma scale <12	41/227 (18)	118/237 (50)	<0.001	10.0%
Oxygen saturation (%)	93 (90 to 96)	92 (88 to 95)	0.01	26.5%
Oxygen saturation <90% †	36/173 (21)	66/205 (32)	0.015	26.5%
*Biological variables (blood)*				
Leucocytes (G/L)	12,128 ± 8,914	13,834 ± 6,902	0.017	3.2%
Plasma sodium (mmol/L)	137 ± 9	138 ± 11	0.08	1.8%
Hypernatremia (>145 mmol/L)	33/250 (13)	49/255 (19)	0.07	1.8%
Creatinine (μmol/L)	119 ± 66	155 ± 85	<0.001	1.9%
Creatinine >120 μmol/L	93/247 (38)	155//257 (64)	<0.001	1.9%
Bicarbonates (mmol/L)	24.6 ± 4.0	22.1 ± 4.5	<0.001	3.5%
Arterial pH	7.42 ± 0.05	7.39 ± 0.10	0.006	89.5%
Infection	93/248 (37)	79/259 (30)	0.11	1.4%
Therapeutic cooling	181/252 (72)	195/262 (74)	0.55	0%
Admitted to ICU	9/252 (4)	26/262 (10)	0.005	0%
Critically ill	47/247 (19)	134/259 (52)	<0.001	1.6%
Risk score	8 (6 to 11)	12 (8 to 16)	<0.001	15.9%
Risk score categories §				
- low	63/228 (28)	27/238 (11)		
- intermediate	128/228 (56)	101/238 (42)	<0.001	9.3%
- high	37/228 (16)	110/238 (46)		

Data are mean + SD, median [25 to 75 interquartile, or number (%).

†, measured using pulse oximetry. *, % of missing values were provided for the global population (no significant differences between the two groups). ¶ despite some missing values precludes exact calculation of the risk score, the risk score category could be allocated to 34 patients because these missing values were not able to modify it.

AII I: angiotensin II inhibitors; CEI: converting enzyme inhibitor; SBP: systolic arterial blood pressure.

**Table 2:** Variables associated with an increased cardiac troponin I (n = 448)

Variables *	Odds ratio (95 percent CI)	***P* value**
Heart rate > 110 bpm	3.59 (2.35 to 5.49)	<0.001
Glasgow coma scale < 12	3.11 (1.95 to 4.95)	<0.001
Creatinine > 120 μmol.L^-1^	1.85 (1.21 to 2.81)	0.004
Self-reported coronary artery disease	1.74 (1.09 to 2.76)	0.02

*, Selection using forward stepwise logistic regression. CI: confidence interval.

**Figure 3:**
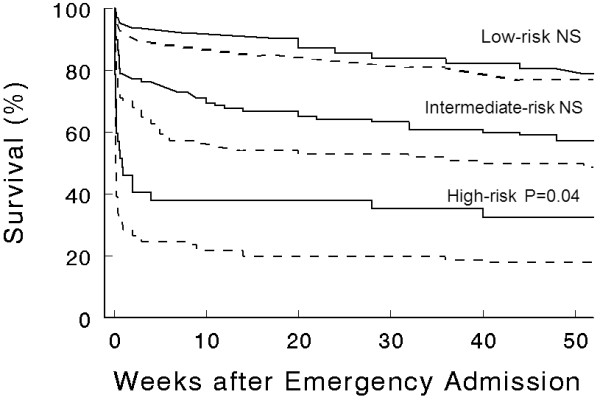
**Kaplan-Meier Survival Curves in patients with or without elevation of cardiac troponin I according to the risk score categories (low-, intermediate-, and high-risk sub-groups, n = 90, 229 and 147 respectively), as previously defined **[11]. *P*-values refer to difference between groups with or without elevation of cTnI. NS, non significant.

Because of missing values, the heatstroke risk score could be calculated in only 432 (84%) patients. In 34 (7%) patients some missing values precluded exact calculation of the risk score, but the risk score category could be allocated because these missing values were not able to modify it. Thus, 147 (31%) patients were in the low-risk group, 229 (49%) in the intermediate-risk group, and 90 (19%) in the high-risk group. When patients were stratified according to this risk score, survival rate was significantly lower in patients with elevated cTnI only in the high-risk group (Figure 3).

**Figure 4:**
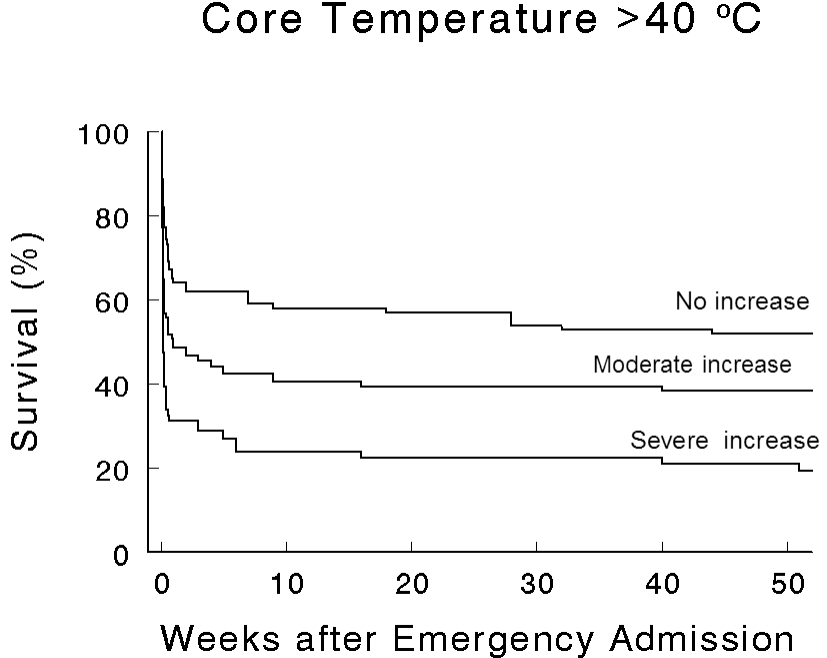
**Kaplan-Meier Survival Curves in the subgroup of patients with a core temperature >40°C (n = 281), according to troponin elevation ranges**. Without elevation of cardiac troponin I (cTnI, n = 105), with moderate increase (abnormality threshold to 1.5 ng.mL^-1^, n = 102) and severe increase in cTnI (>1.5 ng.mL^-1^, n = 74). All differences were significant (*P *< 0.05).

When considering the subgroup of patients with a core temperature >40°C, survival rate was significantly lower in patients with elevated cTnI (Figure 4).

## Discussion

In this large cohort study, we observed that an elevation of cTnI was very frequent during heat-related illness (52%). Nineteen percent of these patients had a severe increase in cTnI suggesting severe myocardial damage. An elevation of cTnI was an independent prognostic factor, particularly in patients with a high risk of death. The inclusion criteria used in our study requires some comments as in contrast to previous studies we included patients with a core temperature >38.5°C rather than >40°C or even 40.6°C. Our main argument was that considerable excess deaths were observed, even in patients without very high core temperatures, mainly because the population involved was aged and frail. Also because cooling was applied before reaching the hospital in at least 15% of these patients, the maximum temperature recorded may not reflect the maximum core temperature reached. Therefore, from an ED's point of view experiencing unusual massive arriving of patients with heat-related diseases, we rather think that the usual criteria for heatstroke (core temperature above 40°C and central nervous system involvement) which remains valid in exertional heatstroke occurring in young individuals should no longer be retained during classic heatstroke occurring during a heat wave, which represents a catastrophic event with major overcrowding of all health facilities.

Our study has several limitations. First, we were not able to provide data concerning the schedule and/or duration of heat exposition before TnI measurement. However, this may reinforce the potential usefulness of TnI dosage in the real conditions of a heatwave leading many severely ill patients to ED. Second, serial TnI dosages were not available in order to identify secondary acute coronary syndromes. Once again, from the ED's point of view it is of major concern, above all, to quickly identify from the arrival the more severely heat-stressed patients. Third, we did not record electrocardiogram's (ECG) data to correlate with TnI results. Finally, the subgroup of patients with cTnI levels was obviously different from those patients in whom cTnI was not measured. They were older and more dependent as reflected by a lower ADL score. Moreover, several variables indicated that they experienced more severe consequences of heatstroke as reflected by higher body temperature and lower Glasgow coma scale. Therefore, the high incidence of elevated cTnI observed might have been lower in the global population [11]. In contrast, elevation of cTnI has been observed in all patients with severe heatstroke admitted to an ICU during the same period [15], and in 74% of our patients either admitted to an ICU or who should have been admitted into an ICU, according to the expert panel.

cTnI is considered a highly sensitive and specific biomarker of myocardial damage. In the case of myocardial injury, the cytosolic pool of cTnI is released first, followed by a more protracted release from cTnI bound to deteriorating myofilaments. cTnI release is not only encountered during acute coronary syndromes but has also been reported in various pathological conditions such as septic shock [20], pulmonary embolism [21] and severe head trauma [22] including brain death [23], and hemorrhagic shock [24]. However, it has been always considered to reflect some degree of myocardial damage, although the significance of this myocardial damage, from a pathophysiological or a prognostic point of view remains debatable. An increase in cTnI is known to be due to either reversible or irreversible myocardial damage as well as ventricular strain [20]. Very few studies reported an increase in cTnI during either exertional or classic heatstroke [11-15]. Pease *et al*. have recently reported that cTnI was elevated in all 22 patients with severe heatstroke admitted to their ICU and that the increase was more pronounced in non-survivors than in survivors (7.4 vs 1.1 ng.mL^-1^, *P *< 0.01) [15]. In the study reported by Pease *et al*., as well as in our study, the precise mechanism involved in cTnI release remained uncertain in the absence of coronary angiography [15]. This increase in cTnI is not surprising since cardiac abnormalities during heatstroke have been previously reported [1,25-27]. Akhtar *et al*. [27] observed electrocardiographic abnormalities compatible with myocardial ischemia in 21% of patients with heat stroke who required active cooling, while Al-Harthi *et al*. [26] using echochardiography reported regional wall motion abnormalities in 18% of cases in the same conditions. Moreover, heatstroke is known to induce thrombogenesis as part of the promotion of coagulation cascade and inflammatory process [28]. It is likely that the increase in cTnI observed in our cohort could be explained by different pathophysiological mechanisms. A moderate increase could mostly reflect moderate myocardial damage and/or ventricular strain, as previously noted in septic and hemorrhagic shocks [20,24], while a severe increase could be more frequently associated with true acute coronary events. During severe sepsis, the direct myocardial cytotoxic effects of cytokines, reactive oxygen species, and endotoxins have also been proposed as a possible mechanism of cTnI release [20]. It should be pointed out that heatstroke is associated with a severe sepsis-like syndrome and that multiple organ failure is also currently observed in severe heatstroke [1]. Lastly, myocardial damage could also be neurally-mediated through abnormal autonomic nervous system activity, as previously observed during subarachnoid hemorrhage and brain death [23,29,30]. Heatstroke is well known to induce neurological impairment and the association we observed between cTnI release and low Glasgow coma scale may reflect either the severity of heatstroke or the presence of neurological damage. Further studies are required to confirm these hypotheses. Although the mechanisms of cTnI release during heatstroke is not presently established, cTnI is obviously an indicator of myocardial damage and is clearly associated with a poor outcome.

Four variables were independently associated with an increase in cTnI. Three of these variables indicate a severe heatstroke (heart rate, Glasgow coma scale, and increased serum creatinine) and the last one was a pre-existing self-reported coronary artery disease. Surprisingly, body temperature was not retained by the logistic model. However, when considering only patients with core temperature >40°C, elevated cTnI was still associated with a poor outcome. Although a causal link cannot be demonstrated in this observational study, it should be pointed out that a high heart rate indicates a considerable heart strain in the clinical conditions of heatstroke and that an elevated serum creatinine level might reflect global dehydration. The role of renal insufficiency in cTnI elevation has been largely discussed. It is now widely accepted that cTnI release is not caused by renal insufficiency although a decreased clearance of released cTnI might further raise its serum levels and/or prolong the time it remains measurable [30]. In fact, the increased cTnI observed in patients with renal insufficiency is likely to be the result of occult myocardial damage. These cTnI elevations are strongly associated with a poor outcome. The fact that a previous coronary artery disease was not a very important prognostic factor (Table 2) suggests that elevation of cTnI can occur in the absence of acute coronary syndromes during heatstroke, as previously suggested in septic and hemorrhagic shock [20,24].

In our core study [11], we demonstrated that the risk of death in heat-related illnesses occurring during a heat wave is predicted by the presence of 11 variables which either indicate the severity of heatstroke (core temperature, systolic arterial pressure, consciousness, leucocyte count), a greater susceptibility to heatstroke (age, pre-existing disease such as cancer, cardiac disease, or chronic medication with diuretics), or both (transportation to hospital by ambulance), or heatstroke complication (pulmonary or bloodstream infection). We have derived a risk score by combining points for each of these features available at admission, which accurately classify patients into subgroups at low, intermediate, and high risk for death. Taken into account this risk score and using the Cox regression model, we observed that only a severe increase in cTnI was an independent prognostic factor (Table 3). When patients were analyzed according to their risk score, elevation of cTnI significantly modified the one-year survival only in patients of the high-risk group (Figure 3).

**Table 3:** Multivariate Cox proportional-hazards analysis predicting death (n = 432)

Variables	Hazard ratio (95% CI)	***P *** **value**
**Risk score**		
- low	1	-
- intermediate	2.46 (1.50 to 4.02)	<0.001
- high	6.48 (3.92 to 10.71)	<0.001
**Elevation of CTni**		
- no increase	1	-
- moderate increase	1.28 (0.94 to 1.73)	0.12
- severe increase	1.87 (1.33 to 2.93)	<0.001

CI: confidence interval.

The health consequences of an heat wave may be catastrophic as shown by previous recent experiences [2-4,8-13]. For this reason considerable efforts have been made to identify climatic factors that predict the occurrence of heat wave, individual factors that favor the occurrence of heatstroke during an heat wave, or even factors that may alert the emergency departments for prehospital heat-related excess mortality [31]. The risk score developed in our core study provides a useful tool for the emergency team allowing better allocation of therapeutic options, including access to the ICU. Although many of the patients in this study were probably not good candidates for admission to the ICU either because of old age and/or co-morbidities or reduced autonomy, we think that the very low proportion of patients finally admitted (5%) indicates a catastrophic event with considerable overwhelming health capacities, in particular the availability of ICU beds. Our present study suggests that cTnI levels should be measured in these patients, particularly those with a high risk of death, and that only a severe increase in cTnI (>1.5 ng.mL-1) should be considered as indicating a worse prognosis.

## Conclusions

In a large cohort of patients with environmental heat-related illness occurring during the August 2003 heat wave in France, we observed a high proportion of patients with elevated cTnI and this elevation was shown to be an independent risk factor.

## Key messages

• During heatwaves, heat-related illnesses presenting to the emergency department are associated with high mortality and morbidity, especially in the elderly.

• Early identification of prognostic variables in emergency room is essential to determining the therapeutic priorities.

• Troponin I is frequently elevated in patients with non-exertional heat-related illnesses.

• Elevated cTnI is an independent prognostic factor, particularly among high risk heatstroke patients.

## Abbreviations

ADL: activities of daily living; cTnI: cardiac troponin I; ED: emergency department; ICU: intensive care unit; ROC: receiver-operating characteristic; SD: standard deviation

## Competing interests

The authors declare that they have no competing interests.

## Authors' contributions

PHa participated in the conception and design of the study, in the acquisition, analysis and interpretation of data, and was involved in drafting the manuscript. BD, AS, SD, AP, PHe, BM, MA and NT were involved in the acquisition of data in their respective emergency departments. SC reviewed the biological data. YLM participated in statistical analysis. BR participated in the conception and design of the study, statistical analysis and interpretation of data, and in drafting the manuscript.
